# Revolutionizing antimicrobial stewardship, infection prevention, and public health with artificial intelligence: the middle path

**DOI:** 10.1017/ash.2023.494

**Published:** 2023-12-01

**Authors:** Alexandre R. Marra, Bradley J. Langford, Priya Nori, Gonzalo Bearman

**Affiliations:** 1 Hospital Israelita Albert Einstein, São Paulo, Brazil; 2 Department of Internal Medicine, University of Iowa Carver College of Medicine, Iowa City, IA, USA; 3 Dalla Lana School of Public Health, University of Toronto, Toronto, ON, Canada; 4 Hotel Dieu Shaver Health and Rehabilitation Centre, St. Catharines, ON, Canada; 5 Division of Infectious Diseases, Department of Medicine, Montefiore Health System, Albert Einstein College of Medicine, Bronx, NY, USA; 6 Division of Infectious Diseases, Virginia Commonwealth University Health, Virginia Commonwealth University, Richmond, VA, USA

“Beware; for I am fearless, and therefore powerful.”

— Mary Shelley, *Frankenstein*

## Introduction

**Figure 1.**
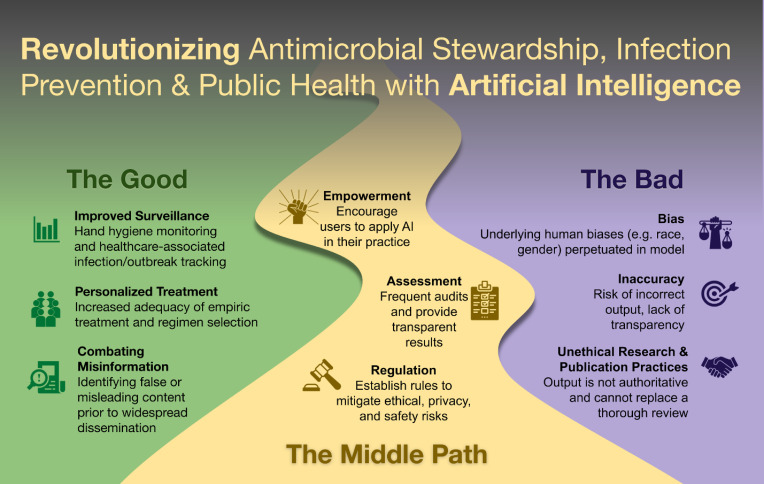
Revolutionizing antimicrobial stewardship, infection prevention, and public health with artificial intelligence.

Artificial intelligence (AI) is the defining technology of our generation, effectively hacking the operating system of our civilization.^1^ The rapid expansion of AI in medicine holds great promise for enhancing the daily practice of healthcare providers. However, as with any emerging technology, important ethical and logistical challenges must be addressed to ensure its safe and effective implementation. The well-established Belmont principles that traditionally apply to Medicine, including autonomy, beneficence, nonmaleficence, and justice, must be extended to AI systems in Medicine.^2^ Expanding these principles to AI systems in health care underscores the significance of autonomy, allowing patients and providers to make informed decisions guided by AI insights. Beneficence takes on new dimensions as AI aids in delivering more precise and personalized care, maximizing patient outcomes. Nonmaleficence remains pivotal, emphasizing the importance of AI systems avoiding harm directly and indirectly. Finally, justice must be to offer fair access and to support social justice.^2^ In this commentary, we explore the application of AI in infection prevention, antimicrobial stewardship, and public health and focus on mitigating its risks (Figure 1).

## The Good: Transforming Antimicrobial Stewardship and Healthcare Epidemiology: The Artificial Intelligence Revolution

1. **How can AI Revolutionize Infection Prevention and Healthcare Epidemiology? Improved surveillance and heightened vigilance for adverse events**

AI, encompassing machine learning and deep learning, offers remarkable capabilities in analyzing and learning from vast amounts of data^3,4^ which can be used to advance the fields of infection prevention and healthcare epidemiology. By integrating machine learning algorithms and video processing, AI can enhance surveillance and the accuracy of hand hygiene compliance monitoring.^5^ Furthermore, AI algorithms can analyze electronic health records (EHRs) and surgical videos to identify patterns predictive of surgical site infections. This enables early detection and timely intervention and reduces the risk of complications.^6^ AI can also facilitate predictive modeling of healthcare associated infections and outbreaks, enabling hospitals to prioritize infection prevention efforts and allocate resources effectively. The integration of AI technologies in infection prevention and healthcare epidemiology has the potential to revolutionize the way health care is delivered. However, this paradigm shift could also raise concerns about the potential displacement or alteration of roles traditionally performed by microbiology technicians, infection prevention and control practitioners, and antimicrobial stewardship clinicians. As AI is increasingly employed to automate laboratory result analysis, predict infection patterns, or recommend treatment strategies, it becomes crucial to address the ethical and workforce implications associated with these changes. Although the displacement of technical duties is inevitable, this may free up time for tasks involving strategic planning (eg, identification and evaluation of novel antimicrobial stewardship and infection prevention initiatives) and human interaction (eg, participating in handshake stewardship). Further, all these applications are in full alignment with the goals of infection prevention, that is, to improve patient outcomes by preventing the spread of infections and optimize healthcare practices.

1. **How can AI revolutionize Antimicrobial Stewardship? Personalized treatment and improved outpatient practices**

AI enhances treatment decisions by providing individualized, real-time recommendations to healthcare providers on optimal antimicrobial treatment. By analyzing patient data and considering factors such as prior antimicrobial use and culture and susceptibility data, AI algorithms further guide clinicians in determining the likelihood of infection, selecting the most appropriate empiric and targeted regimens, provide dose optimization, and minimize the risk of resistance development.^7–10^ The integration of standard operating procedures, analytic tools, data types, and quality control into a laboratory data warehouse accessed by a large language model will create new possibilities for improving clinical microbiology laboratory practices.^11^ Additionally, AI can aid in the prediction of antimicrobial resistance patterns directly from mass spectra profiles compared to traditional laboratory-based susceptibility testing.^12^ Collaboration between healthcare personnel and AI systems requires a mutual understanding of roles and responsibilities. Efforts to ensure that microbiology technicians, infection prevention and control practitioners, and antimicrobial stewardship clinicians are equipped to work alongside AI technologies, leveraging their expertise in tandem with AI insights, can optimize the potential benefits while minimizing potential disruptions.

1. **How can AI improve public health? Combating misinformation, enhancing surveillance, and streamlining patient care and humanitarian aid**

AI also has positive implications for public health. AI systems may potentially combat vaccine misinformation and other medical inaccuracies by analyzing large data sets and identifying false or misleading content prior to its widespread dissemination. AI systems could also be deployed to analyze data from social media and other sources in real time, enabling early detection of health threats and improving response times. At the patient care level, AI solutions integrated within EHRs, incorporating natural language processing, enable the efficient triage of patients reporting positive results from SARS-CoV-2 tests taken at home. This integration leads to reduced time required to respond to a positive test result and increases the probability of receiving an antiviral prescription within the critical 5-day treatment time frame.^13^ Additionally, at the population level, AI optimizes the delivery of humanitarian aid by analyzing data on population density, infrastructure, and resources availability, ensuring aid reaches the most affected areas promptly.^14^

## The Bad: If Technology Executives fear AI, So Should We

A statement by >350 tech executives released in May 2023 summarizes the imminent public health threat of AI: *“Mitigating the risk of extinction from AI should be a global priority alongside other societal-scale risks such as pandemics and nuclear war.”* This followed another high-profile letter signed by executives of Apple and Tesla calling for a 6-month moratorium on the development of advanced AI systems until we have more robust processes to keep them in check.^15^

In June 2023, *Evgeny Morozov,* a writer and researcher, who studies political and social implications of technology espoused numerous warnings in a New York Times opinion piece entitled “The true threat of AI.” While not specifically about health care, Morozov describes AI’s vulnerability to the market’s demands for profits over improving the lives of people and that AI undermines our civic virtues and amplifies trends we already dislike. For instance, in health care, AI algorithms based on ever-increasing sets of patient-level data will become fixated on efficiency and profits over value and further exacerbate an epidemic of healthcare worker burnout and moral injury. Like other emerging technologies of the past, AI can be coopted by “bad actors,” whose victims are real, everyday people (Theranos).^15^

1. **Patient harm caused by biases**

All applications of AI rely on individual patient-level data, which ought to be safe and protected. When patients agree to receive health care within our institutions, they are not necessarily consenting to use of this data for purposes outside of individualized patient care. Informed consent is mandatory for research involving human subjects, but somehow use of patient data for AI applications is a “work around” for the acquisition and use of protected health information.^16^

Moreover, large data sets utilized by AI depend on the information fed into the system which can be inaccurate and can contain harmful biases. Of greatest concern is EHR biases based on race, ethnicity, gender, socioeconomic status, education level, and other social determinants of health which can be input into AI data sets and may serve to perpetuate and amplify biases, causing significant patient harm. For example, algorithms trained on healthcare expenditure data in which Black patients systematically received less care than their White counterparts can lead to underestimating the level of risk of Black individuals. If such data are incorporated into AI models to manage or prevent infection, there is a risk of embedding and inadvertently reinforcing racism in AI-informed practice, leading to continued inequitable patient outcomes. Those left out by current structural barriers to optimal health care will remain at risk. For example, an AI-powered diagnostic tool trained on historical data that underrepresents certain demographic groups may lead to misdiagnoses or inadequate medical recommendations for individuals belonging to those groups. Biases within AI systems may also result from the lack of diversity and representation among the developers and data scientists involved in their creation. When the development teams lack diverse perspectives and experiences, it becomes more challenging to identify and rectify biases in AI models. This lack of diversity can contribute to a feedback loop where biased AI perpetuates the same disparities it was intended to mitigate.^17^ Concerns also exist about transparency and trust in AI tools. Understanding the sources of training and validation data is fundamental for confidence in large language model capabilities. The “black box” nature of these models further exacerbates these concerns, as users are unaware of AI system biases, thereby eroding public trust. This is particularly relevant when AI predictions are incorrect.

1. **Accuracy: can we trust what we get back from Generative AI Models?**

ChatGPT-4 is neither sufficiently mature to formally diagnose patient conditions nor replace health professionals.^11,18^ Large language models produce outputs that are coherent but can be confidently incorrect or nonsensical. For clinical microbiology and infectious diseases, large language model outputs are of good quality, but without identifiable sources and references that are often nonexistent or “hallucinated.”^11^

In medical practice, diagnoses commonly lack a definitive confirmatory test, relying instead on clinicians reaching a diagnostic consensus from the clinical presentation and available laboratory analyses. Although AI is valuable in objectively diagnosing conditions with clear numerical indicators, such as acute kidney injury, determining conditions like ventilator associated pneumonia is more complex. Assessing the accuracy of AI is challenging because clinical diagnosis often involves interpreting imprecise and nonnumerical data, with no definitive tests available.^18^

AI system must have an audit trail that can be reviewed such that its performance can be continuously monitored, such as *algorithmovigilance* ^19^ describes postdeployment monitoring of AI for serious failure, performance drift, “off label” use, and other problematic developments, in much the same way that drugs are subject to postmarket pharmacovigilance.^2^

1. **Negative implications for research and publication**

In addition to the concerns related to biases and transparency, the use of ChatGPT-4 and similar language models can have negative implications for research and publication. Although these models can be helpful in generating text and providing information, they should not be considered as authoritative sources for academic or scientific research. One potential and relevant issue is the risk of plagiarism. Since these language models can generate coherent and seemingly well-informed responses, there is a possibility of directly copying the model’s outputs without proper citation or attribution. This can undermine the integrity of academic work and intellectual property rights.^4,20^

Researchers should be cautious about overreliance on AI language models and instead cultivate their own expertise, conduct through literature reviews, and engage in scholarly discourse. Scientific originality and transparency must prevail. Although the AI language models can provide a quick response, it lacks the ability to engage in meaningful discussions, consider alternative viewpoints, or evaluate the quality and validity of sources.^4^

Another concern is the potential for misinformation propagation. Although efforts are made to train AI models on reliable and reputable sources, there is still a risk of incorporating biased or incorrect information into the model’s responses. Users who blindly trust the outputs of the AI models may inadvertently spread misinformation, especially if they fail to critically evaluate and fact-check the content.^17^ ChatGPT-4 and other similar models are increasingly used in research to generate code for programming languages such as Python and R. While often time-saving, the output from such large language models could contain malicious code that can then be inadvertently installed on the user’s computer.^2^

To mitigate these risks, clear guidelines and ethical standards for the use of AI language models in research and publications are urgently required.^17,20^ Researchers should be encouraged to use these AI models as tools to support their work rather than replacing rigorous academic practices. Academic institutions and publishers must play a role by providing guidance on responsible AI usage, promoting proper citation and attribution practices, and emphasizing the importance of critical thinking and independent analysis.

1. **Potential to supersede human oversight and worse: the more information we feed it, the more we help to refine it and fuel our “enemy”**

One significant concern regarding AI language models, including the ChatGPT-4, it is the potential for these systems to supersede human oversight.^1,15^ As these models continue to evolve and improve through the accumulation of vast amounts of data, they may become increasingly autonomous and independent from human control.^1^ This raises important ethical and societal questions about the extent to which we should rely on AI systems to make decisions or provide information without human intervention.^17,20^

As previously mentioned, allowing AI language models to operate with minimal human oversight may lead to unintended consequences.^1^ The US Food and Drug Administration plays a leading role in global discussions on regulatory oversight for AI-based medical tools, establishing regulations for emerging technologies in the medical field that utilize AI.^17^

Furthermore, the continuous refinement and performance of AI language models occur with ongoing data inputs, eventually minimizing the need for human oversight. The loss of human oversight is a unified concern and warning from individuals such as technology executives, who are all too familiar with the pros and cons of “disruptive technology.”

## The Voice of Reason: The Middle Path

“Avoiding extremes, the wise gain the experience of the Middle Path which produces insight, calms, and leads to higher knowledge, enlightenment.” – The Buddha

Finding a middle ground in the development and deployment of AI is critically important to harness its potential and mitigate both risks and ethical concerns. First, it is crucial to ensure that the data sets used to train AI models are diverse, broadly representative, and free from systemic biases. Clinicians should advocate for, and developers should focus on addressing biases, improving fairness, and addressing potential risks through regular updates and advancement in the models.^15^ Regular audits and assessments should be conducted to detect and mitigate any biases that may emerge in AI systems.^17^ Transparency should be encouraged by *explainable AI*, which can help clinicians and patients peer inside the “black box” and foster trust in AI strategies in health care.^8^ Additionally, promoting diversity and inclusivity in AI development teams can help mitigate biases and enable the creation of more equitable and fair AI applications.

Empowering users to employ and navigate AI language models is key to their successful adaptation. This requires user-friendly interfaces. With ease of use, AI systems may be immediately employed in both antimicrobial stewardship and infection prevention. AI can predict anti-infective drug activity, drug–target interactions, and therapeutic design.^21^ Antimicrobial stewards and infection preventionists should be encouraged to seek opportunities to apply AI for daily functions (eg, becoming familiar with generative AI platforms such as ChatGPT and Consensus^22^) and larger pursuits (eg, idea suggestions for a scientific paper or presentation, pursuing research funding opportunities, and developing task forces to address AI application) to improve productivity in their respective fields.^21,23^ Last, ongoing research and innovation must focus on addressing the limitations and challenges of AI language models. Exploring novel techniques for bias mitigation and ethical decision-making can pave the way for more responsible and beneficial AI systems.^1^

To summarize, AI language models require a proactive multifaceted approach that combines regulatory measures, user empowerment and equity, collaboration, iterative improvements, public trust-building, ease of use, and continuous research to mitigate errors. By systematically implementing these aspects, we can navigate the complexities of AI technology and ensure its responsible adoption to revolutionize antimicrobial stewardship, infection prevention, and public health.
